# The Kinome of Pacific Oyster *Crassostrea gigas*, Its Expression during Development and in Response to Environmental Factors

**DOI:** 10.1371/journal.pone.0155435

**Published:** 2016-05-27

**Authors:** Yanouk Epelboin, Laure Quintric, Eric Guévélou, Pierre Boudry, Vianney Pichereau, Charlotte Corporeau

**Affiliations:** 1 Ifremer, UMR 6539 CNRS/UBO/IRD/Ifremer, Laboratoire des sciences de l’Environnement Marin, Plouzané, France; 2 Ifremer, Service Ressources Informatiques et Communications, Plouzané, France; 3 UBO, UMR 6539 CNRS/UBO/IRD/Ifremer, Laboratoire des sciences de l’Environnement Marin, Plouzané, France; Chang Gung University, TAIWAN

## Abstract

Oysters play an important role in estuarine and coastal marine habitats, where the majority of humans live. In these ecosystems, environmental degradation is substantial, and oysters must cope with highly dynamic and stressful environmental constraints during their lives in the intertidal zone. The availability of the genome sequence of the Pacific oyster *Crassostrea gigas* represents a unique opportunity for a comprehensive assessment of the signal transduction pathways that the species has developed to deal with this unique habitat. We performed an *in silico* analysis to identify, annotate and classify protein kinases in *C*. *gigas*, according to their kinase domain taxonomy classification, and compared with kinome already described in other animal species. The *C*. *gigas* kinome consists of 371 protein kinases, making it closely related to the sea urchin kinome, which has 353 protein kinases. The absence of gene redundancy in some groups of the *C*. *gigas* kinome may simplify functional studies of protein kinases. Through data mining of transcriptomes in *C*. *gigas*, we identified part of the kinome which may be central during development and may play a role in response to various environmental factors. Overall, this work contributes to a better understanding of key sensing pathways that may be central for adaptation to a highly dynamic marine environment.

## Introduction

The Pacific oyster *Crassostrea gigas* is a sessile marine invertebrate living in estuarine and intertidal zones and is therefore exposed to dramatic environmental fluctuations. *C*. *gigas* is one of the model species for aquaculture worldwide, but is also classified as invasive in many countries, reflecting its ability to establish populations in a broad range of environmental conditions. Oysters must deploy multiple systems to cope with environmental changes, by adapting their metabolic activities and transmitting danger signals to their defense systems [1] [2] [3]. Research dedicated to this species has grown significantly in recent decades [4] and *C*. *gigas* is the first marine sessile bivalve for which the genome has been completely sequenced [5]. Nevertheless, a universal understanding of its regulatory functions and interactions is still lacking.

Eukaryotes cope with their environments using a variety of mechanisms at different levels, including physiological, biochemical and molecular processes. Among these processes, post-translational modifications (PTM) have been described as one of the most important mechanisms for activating, modifying or suppressing protein functions and for increasing the proteome functional diversity [6]. PTMs change protein properties either by proteolytic cleavage or by addition of a modifying group to one or several amino acids [7]. Protein modifications include processes such as acetylation [8], methylation [9], or phosphorylation [10]. Protein phosphorylation is known to play a central role in regulating the basic functions of all eukaryotes, including DNA replication, cell cycle control, cytoskeletal rearrangement, cell movement, gene transcription, protein translation, apoptosis, differentiation and energy metabolism [11]. This process is also required to mediate defense responses and complex interactions with the external environment. The key enzymes that regulate protein phosphorylation and control cell signal transduction are protein kinases. In humans, deregulation of protein kinases is often associated with pathological states, and mutations in kinase genes are known to be involved in apoptosis, inflammation, diabetes and cancer [12]. Based on genomic data from some model species, protein kinases were identified as the largest superfamily of enzymes, representing about 2% of the whole proteome [13]. They act by phosphorylating serine, threonine or tyrosine residues, to induce structural and functional modifications of the target proteins [14], and modifying downstream target enzymatic activities, cellular localization and/or association with regulatory proteins and factors.

The characterization of the kinome involves the identification and classification of protein kinases, and has been performed previously in some species ranging from yeast to human (results available at www.kinase.com) [15] [16] [17] [18]. A strong positive linear correlation between kinome and proteome sizes has been described in model species, including human *Homo sapiens*, the nematode *Caenorhabditis elegans*, the fly *Drosophila melanogaster*, the amoeba *Dictyostelium discoideum*, and the yeast *Saccharomyces cerevisiae* [19]. Protein kinases can be divided into two superfamilies based on the 250–300 amino acid sequences of their catalytic domains and their kinase activity: (i) eukaryotic protein kinases (ePK) with a conserved catalytic domain, and (ii) atypical protein kinases (aPKs) which have no structural similarity with ePKs, but have been shown experimentally to display kinase activity [15].

The ePKs can be split into nine groups: AGC (cAMP-dependent protein kinase/protein kinase G/protein kinase C extended), CAMK (Calcium/Calmodulin regulated Kinase), CMGC (Cyclin-dependent Kinase and other close relatives), CK1 (Cell or Casein Kinase I), RGC (Receptor Guanylate Cyclase), TK (Protein Tyrosine Kinase), TKL (Tyrosine Kinase Like), STE (involved in mitogen-activated protein kinase cascade), and "others" characterized by lower sequence similarities [20]. The AGC group contains protein kinases that are activated by second messengers, such as the PKA (cAMP-dependent Protein Kinase), PKG (cGMP-dependent Protein Kinase) or PKC (Protein Kinase C) families [21]. The CAMK group phosphorylates serine and threonine residues preferentially near basic amino acids [22]. The CMGC group mainly contains CDK (Cyclin-Dependent Kinase) families involved in cell cycle control and MAPK (Mitogen-Activated Protein Kinase) families involved in signal transduction [23]. CK1 is a small group known to preferentially phosphorylate acidic regions [23]. The RGC group includes receptors with an active guanylate cyclase domain that generates cyclic GMP (Guanosine Monophosphate) [24]. Kinases in the TK group phosphorylate specifically tyrosine residues, and play a role in signal transduction [25]. TKL are highly similar to TK and phosphorylate serine and threonine residues [13]. Receptor protein kinases in the TK and TKL groups sense environmental stimuli and transfer signals from the cell membrane to the nucleus, through the regulation of kinases that belong to the STE group. The STE group contains protein kinases involved in signal transduction upstream of the MAPK family [26] [27].

Atypical protein kinases (aPKs) have been identified by biochemical methods and include proteins known to be involved in the phosphorylation-mediated regulation of a wide variety of cellular processes [28]. Many of these aPKs have been shown to bare significant structural homology to ePKs despite their lack of sequence similarity, while others are structurally distinct [13]. Some aPK families are conserved across numerous species, including prokaryotes, while others are restricted to metazoans.

To date, little is known about the classification of protein kinases in lophotrochozoa, a little-studied clade of bilaterian animals that includes marine bivalves. The recent availability of the *C*. *gigas* genome [5] represents a unique opportunity for a comprehensive study of the kinome in a species adapted to life in the intertidal zone, a stressful and highly dynamic environment. In the present article, we describe the first genome-wide analysis of *C*. *gigas* protein kinases. We identified, annotated and classified protein kinases in *C*. *gigas* according to their kinase domain taxonomy, and compared the resulting kinome to those of other species.

Activation of protein kinases is mainly governed by post-translational modification, such as rapid (within minutes) phosphorylation/dephosphorylation processes. However, long- term (within hours) activation of kinases has been shown to induce modification of their gene expression [29]. Analyses of mRNA expression levels of protein kinases has already been done in widely-studied organism from yeast to humans, offering insight into signaling in unicellular and multicellular organisms [16]. Data mining of transcriptome data [5] allowed us to analyze specific expression of protein kinases during early development and in response to environmental factors. Our study sheds light on the molecular signals that might be involved in the adaptation of oysters to their environment.

## Materials and Methods

### Identification of ePKs and aPKs in the *C*. *gigas* genome

The identification of eukaryotic protein kinases (ePKs) and atypical protein kinases (aPKs) was based on predicted proteins of *Crassostrea gigas*, as it was done similarly in *Caenorhabditis elegans* [11], microsporidia species [19] and *Dictyostelium* [30]. *C*. *gigas* predicted proteins were retrieved from version 9 of the complete genome and downloaded from NCBI (http://www.ncbi.nlm.nih.gov/bioproject/PRJNA70283) [5]. Initially, putative protein kinases were detected using a Hidden Markov Model (HMM) profile based on known ePKs, in order to screen the 26,086 predicted *C*. *gigas* proteins. To assess the first selection, we also identified protein kinases using ePKs PFAM (PF00069) [31]. For the final catalog, we compared the annotation as protein kinase (using HMM and PFAM) with the automatic annotation of the proteome [5] using an in-house non-stringent E-value cut-off of 10^−2^. We carefully inspected all annotations and were able to improve the annotation for 9 protein kinases in the proteome.

### Classification

*Crassostrea gigas* ePKs and aPKs were classified into groups and families, based on the similarity of their catalytic domains with other species, including human *Homo sapiens*, mouse *Mus musculus* [32], the sea urchin *Strongylocentrotus purpuratus* [17], the nematode *Caenorhabditis elegans* [11], the fruit fly *Drosophila melanogaster* and the amoeba *Dictyostelium discoideum* [30], all downloaded from Kinbase (http://www.kinase.com/kinbase). Proteins were classified according to existing taxonomy by BLAST search with an E-value cutoff of 10^−10^. Some protein kinases with multiple conserved catalytic domains could be classified into different groups. In this case, multiple sequence alignment was performed to infer a phylogenetic tree to allow us to manually reclassify them into better fitting groups. Information about the 371 selected sequences is provided as supplementary information (S1 Table): Genbank accession number, classification (group/family/subfamily), query definition, best hit name and percentage of identity compared to other species with corresponding E-values.

### Phylogenetic analysis

A phylogenetic tree was built using the *C*. *gigas* ePK domains using the neighbor joining method. Sequences analysis was performed using the maximum likelihood method of PhyML [33]. For the RGC group, a tree was built from protein sequences aligned against the entire PF00069 domain with hmmalign [34], manually refined using Jalview [35], then significant blocks were selected. The oyster RGC phylogenetic tree was inferred with the maximum likelihood method of PhyML with 1000 bootstrap replicates, using the complete set of human, urchin, nematode, fruit fly genes encoding RGC kinases, and insulin-related peptide receptor (EKC21734.1) as outgroup. Both trees were visualized using the Figtree program (http://tree.bio.ed.ac.uk/software/figtree).

### Data mining of available RNA-seq data for *C*. *gigas*

Information regarding down and up-regulation of gene expression was extracted from *C*. *gigas* RNA-seq data (49 bp single-end Illumina reads) available on the NCBI website [5]. Gene expression levels were measured by RPKM (reads per kilobase per million mapped reads) [5]. To minimize the influence of sequencing depth between samples, the total read number was normalized by multiplying a normalization factor [36]. This strategy introduces a scaling factor called Trimmed Mean of M-values (TMM), which aims at representing the “global fold-change” [36].

The RNA-seq data used were obtained from several developmental stages: egg, two cell stage, four cell stage, morula, blastula, gastrula, trochophore, D-larva, umbo larva, pediveliger, and two additional datasets consisting of spat (day 22 post fertilization) and juveniles (day 215 post fertilization) [5]. Genes encoding kinases with expression values < 1 RPKM were considered to be non-expressed.

Additional RNA-seq data, published by the same authors, were obtained from adult oysters subjected to 8 types of environmental factors [5]. Differentially expressed genes were detected using a method [37] which was constructed based on a Poisson distribution in order to avoid the influence of sequencing depth and gene length [5]. Seven oysters were exposed to different temperatures (5, 10, 15, 20, 25, 30 and 35°C) and gills were sampled after 12h and 7 days. Oysters maintained at 20°C were used as the control. Gills were collected at 7 days to evaluate the impact of salinity on 7 oysters (5, 10, 15, 20, 25, 30 and 40 ppm), with a salinity of 30 ppm as control. Fifteen oysters were exposed to air and gills and adductor muscles were collected after 1, 3, 5, 7, 9, 10 and 11 days. Control oysters were placed in aerated seawater. For metal toxicity studies, gills and the digestive glands of 10 oysters exposed to zinc (1 mg/L) were sampled at 0 and 12 h, and after 5, 7, 9 and 13 days. In another experiment, gills and digestive glands were sampled in 4 oysters exposed during 12 h and 9 days to each of the five metals (zinc at 1 mg/L; cadmium at 100μg/L; copper at 100μg/L; lead at 500μg/L; mercury at 20 μg/L). Control oysters were kept in seawater at 20°C and salinity of 30 ppm without metal addition [5].

## Results and Discussion

### Overall description of the first marine bivalve kinome

The genome of *C*. *gigas* was used to construct an *in silico* proteome containing 26,086 single encoded proteins. Among them, we generated a non-redundant set of 371 protein kinases (S1 Table) using the HMM profile and BLAST homology searches in metazoan model organisms. The ePKs were then classified based on the known sequences of the catalytic region, together with features of the non-catalytic accessory domains, since their modular architecture is predominant to define their biological roles even though it is conserved to varying degrees among the ePKs [38] (Table 1).

**Table 1:** Taxonomic distribution of protein kinases (ePKs, aPKs) in various species.

	Oyster	Yeast^a^	Worm^a^	Drosophila^a^	Sea urchin^b^	Human^a^
Group of kinases	*Crassostrea gigas*	*Saccharomyces cerevisiae*	*Caenorhabditis elegans*	*Drosophila melanogaster*	*Strongylocentrotus purpuratus*	*Homo sapiens*
AGC	28	17	30	30	29	63
CAMK	51	21	46	32	50	74
CMGC	39	21	49	33	35	61
CK1	6	4	85	10	6	12
RGC	23	0	27	6	8	5
TK	70	0	90	32	53	90
TKL	40	0	15	17	35	43
STE	28	14	25	18	21	47
Other	77	38	67	45	92	83
aPK	9	15	20	17	24	40
TOTAL Kinases	371	130	454	240	353	518

^a^ data from [16]

^b^ data from [17]

The resulting kinome corresponds to 1.4% of the whole proteome. This result is consistent with the correlation previously observed between kinome size and genome size [19]. Their evolutionary position provides important information on the evolution of kinases in Lophotrochozoa, a relatively distant group of species compared to those for which the kinome had already been characterized. Assignments of *C*. *gigas* ePKs and aPKs to different groups, including data from representative species, are shown in Table 1. In *C*. *gigas* (genome size: 557 Mb), we identified a total of 362 ePKs, a number close to the 329 identified in the sea urchin *S*. *purpuratus* (genome size: 814 Mb) [17] [39], but different from *C*. *elegans* for which 434 ePKs (genome size: 103 Mb) have been discovered [40].

**Fig 1:**
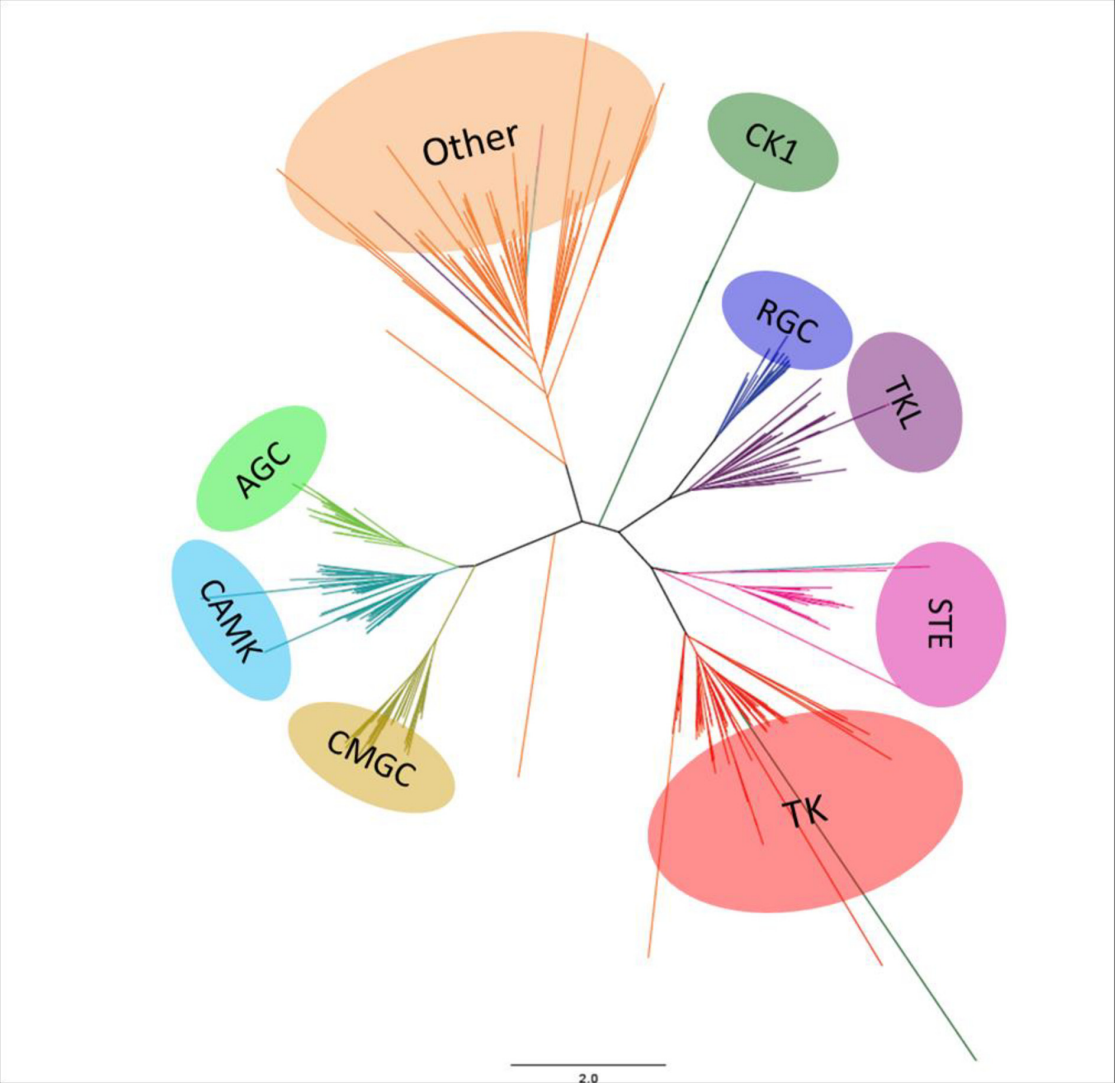
A tree of the *C*. *gigas* ePKs superfamily. Phylogenic analysis of amino acid sequences of *C*. *gigas* ePKs domains by neighbor joining. The tree is visualized with Figtree. The color blocks represent the nine groups of ePKs shown in this tree: AGC (PKA, PKG, PKC containing), CAMK (Ca/Calmodulin-type), CMGC (CDK, MAPK, GSK, CDKL), CK1 (Casein kinases), RGC (receptor guanylate cyclase), TK (tyrosine kinases), TKL (tyrosine kinase-like), STE and Others. The aPK superfamily was excluded from this analysis.

Based on our classification of ePKs and aPKs (S1 Table), we then determined which protein kinases were conserved between *C*. *gigas* and other species, and investigated their biological functions. Phylogenetic analyses with metazoan ePKs were performed to validate the affiliation of each oyster ePK (Fig 1). We showed that all ePKs were distributed into nine distinct classes, as described in almost all available kinomes. Indeed, some fungal species including the yeast *S*. *cerevisiae* have a reduced kinome with the loss of one or more groups (for instance the TK group) [20] [41]. The nine ePK groups are present as a single cluster, although some protein kinases were distributed in the phylogenetic tree into unexpected groups based on the BLAST and HMM-based analyses. This may be due to the inherently imperfect nature of the heuristic methods used to generate phylogenies [17]. Overall, the nine groups of ePKs are well represented, with a distribution similar to sea urchin, worm, drosophila or even human kinomes. The classification of 28 protein kinases remained ambiguous due to low sequence similarity and high BLAST E-values, suggesting that they might correspond to oyster-specific kinases. In *S*. *purpuratus*, 21 protein kinases with no family-level homologs in other organisms were identified as urchin-specific and belong to the Other group [17]. The function of urchin-specific protein kinases is unknown. They were shown to display weak expression during embryo development, thus suggesting that these kinases should be mainly implicated in adult-specific functions [17]. In *C*. *gigas*, another surprising feature of the oyster kinome is the large number of protein kinases that belong to the RGC group. A detailed description of observed similarities between kinomes of *C*. *gigas* and other species for the nine ePK groups, including RGC and the aPKs, is presented below.

### AGC group

Twenty-eight AGC kinases were found in *C*. *gigas*, representing 7.5% of its kinome. This is similar to the number of AGC kinases described in the worm *C*. *elegans* (6.6%) and the sea urchin *S*. *purpuratus* (8.2%), but lower than in Drosophila and human (12.5%) (Table 1). AGC kinases have been well described in humans, with roles in signal transduction networks and an influence on a large range of biological responses [21]. Specifically, they are involved in growth factors, insulin and lipid signaling, and are linked to G protein coupled receptors. In *C*. *gigas*, the AGC group (28 members) contains all the families and subfamilies existing in the metazoan lineage, but has fewer members as compared to human (63 members). Interestingly, several members of the AGC group are represented by a single ortholog, in contrast to humans that have multiple redundant isoforms. *C*. *gigas* has only one protein kinase AKT (Protein kinase B) (while there are three in human and two in sea urchin) and one p70S6K (2 in human; 1 in sea urchin). In mammals, AKT was shown to be a protooncogene regulated by the lipid tyrosine kinase phosphatidylinositol 3 kinase (PI3-K) functioning as a cell survival signal to protect cells from apoptosis, and p70S6K controls the rate of protein translation during growth. Target studies of mRNA expression showed that these functions might be conserved in *C*. *gigas*, because AKT is regulated during gametogenesis [42] and p70S6K is involved in insulin signaling [43]. Insulin receptor, RAS, PI3-K and PDK1, all act as upstream activators of AKT, that is inducing cell survival. Each of these components of the PI3-K/AKT pathway is conserved in *C*. *gigas*, providing a powerful system for understanding cell survival signal in response to stress.

We also identified one animal-specific YANK (yet another novel kinase) (while there are four in human and one in the sea urchin) with unknown function. A detailed classification of AGC in *C*. *gigas* is provided in S1 Table.

### CAMK group

Fifty-one CAMK kinases were identified in *C*. *gigas*, covering all families and subfamilies described in human (74 CAMK) and in the sea urchin (50 CAMK) (Table 1). Calcium-mediated signaling plays crucial roles in vertebrates during fertilization, embryonic development, signal transduction (through MAPK signaling), protein secretion, transport and memory [22] [44]. The *C*. *gigas* kinome includes five MLCK (Myosin Light Chain Kinases), that phosphorylate the regulatory light chain of sarcomeric myosin [45], and four MARK (Microtubule Associated Kinases) that play roles in cytoskeletal organization and microtubule dynamics [46]. A similar number of TSSK (testis-specific serine/threonine-protein kinases) has been identified in *C*. *gigas*. TSSK are known to play an important role in spermatogenesis in humans [47]. In another marine bivalve, the Peruvian scallop *Argopecten purpuratus*, the mRNA of one TSSK was shown to be differentially expressed depending on the maturation stage, sex and tissue analyzed, suggesting a potential function of TSSK in reproductive mechanisms [48].

In the AGC group we identified the catalytic subunit α of the 5’-AMP-activated protein kinase (AMPK). AMPK is an heterotrimeric kinase composed of a catalytic α-subunit and two regulatory subunits, β and γ. Interestingly, each of the AMPK subunits are encoded by a single gene in *C*. *gigas* [42]; the absence of redundancy simplified functional studies of AMPK in this species. The protein kinase AMPK is a key regulator of cell energy metabolism in eukaryotes [49]. As it is the case for almost all kinases, AMPK activation is regulated at the post-translational level through its phosphorylation, and *C*. *gigas* AMPKα has conserved the characteristic threonine 172 active site, as well as the binding domain for regulatory β and γ subunits [50]. In mammals, AMPK is activated by metabolic stresses such as glucose deprivation, oxidative phosphorylation, ischemia, and hypoxia [51] [52]. In *C*. *gigas*, the activation of AMPKα controls metabolism during gametogenesis [42] and is activated in response to environmental-stress, for example 6h of hypoxia [53] or 14 days of exposure to low concentrations of pesticides [54]. In another bivalve, the freshwater mussel *Elliptio complanata*, AMPKα has been proposed as a biomarker of *in situ* short-term contamination [55].

### CMGC group

Thirty nine CMGC in *C*. *gigas* are distributed between all families and subfamilies described in humans (61 members) [13] as well as in the sea urchin (35 members) [17] (Table 1). The CDK (Cyclin-Dependent Kinase) and MAPK (Mitogen-Activated Protein Kinase) families represent around 70% of this group. We identified the three major MAPK cascades in *C*. *gigas* represented by ERK, p38 MAPK and JNK, known to be crucial in cell signal transduction [56]. In another bivalve, the mussel *Mytilus galloprovincialis*, the p38 MAPK and JNK signaling pathways are activated in response to various environmental stressors, such as temperature or heavy metals, leading to regulation of apoptosis [57] [58]. *C*. *gigas* also has five members of the DYRK (Dual specificity Tyrosine Regulated Kinase) family that play key roles in cell proliferation and apoptosis induction in response to stress such as DNA damage [59]. The *C*. *gigas* kinome includes one casein kinase CK2 known to be involved in the response to oxidative stress in *M*. *galloprovincialis* [60] and the regulation of carbohydrate metabolism [61]. In the CMGC group, GSK-3β is present as a single ortholog and might be a key regulator of gonadal development [62], as demonstrated in the Portuguese oyster, *Crassostrea angulata* [63]. In *C*. *gigas*, MAPK signaling is also mainly implied in maintaining metaphase I arrest in oocytes [64].

### CK1 group

The *Crassostrea gigas* kinome contains six proteins of the CK1 group, including four isoforms of casein kinase 1 and two isoforms of TTBK (Tau-tubulin kinase). CK1 and TTBK are involved in diverse cellular processes, including membrane trafficking, circadian rhythm, cell cycle progression, chromosome segregation, apoptosis, cell division, DNA repair, and cellular differentiation [65]. CK1 is the smallest group of protein kinases in most of the species for which the kinome has been identified [66], with four members in budding yeast, and six in human and *C*. *gigas*. Surprisingly, in *C*. *elegans*, 87 CK1 were characterized, and it was hypothesized that this huge diversification might be an adaptation allowing enhanced DNA repair in response to excessive exposure to environmental stressors and mutagens [11].

### RGC group

**Fig 2:**
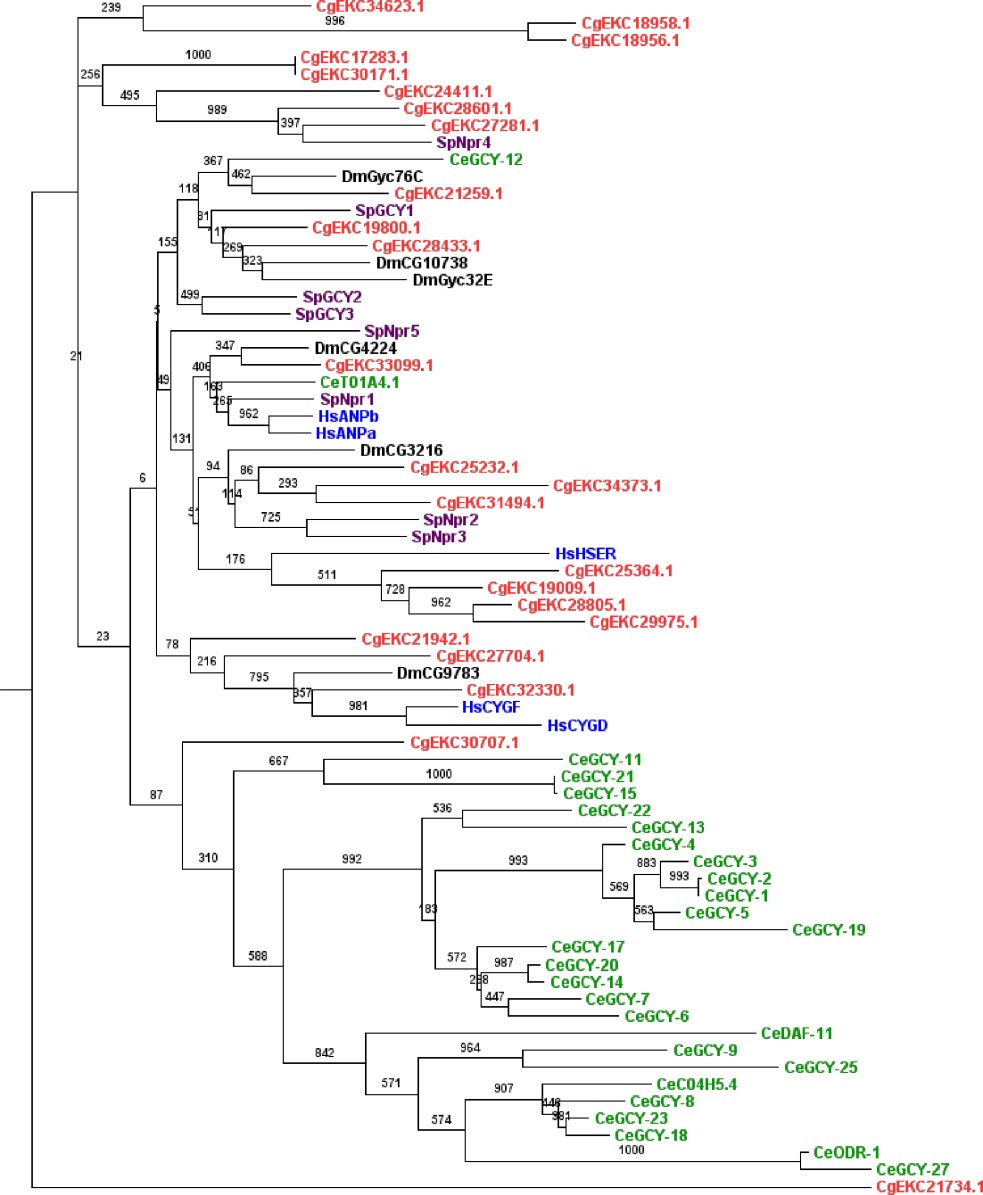
Phylogenetic analysis of *C*. *gigas* RGC kinases. This tree was generated from RGC domain amino acid sequences from several species (Red: *C*. *gigas*, Purple: *S*. *Purpuratus*, Green: *C*. *elegans*, Blue: *H*. *sapiens*, Black: *D*. *melanogaster*), using the PhyML maximum likehood program and visualized with Figtree. A protein kinase from the TK group was used as outgroup (Insulin-related peptide receptor [CgEKC21734.1]). Corresponding maximum parsimony (MP) bootstrap values are shown on each branch.

RGC kinases are single-pass transmembrane receptors and have a catalytically inactive kinase domain, but an active guanylate cyclase domain that catalyzes the synthesis of cyclic GMP from the energy carrier GTP (Guanosine triphosphate) [67]. These receptors are activated by external ligands such as Atrial, Brain Ventricular and C-type Natriuretic Peptides (ANP, BNP, VNP and CNP), which are endocrine hormones involved in cardiovascular and osmoregulation systems in vertebrates [68] [69]. The RGC group is particularly abundant in *C*. *gigas* with 23 protein kinases, i.e. 15 more than in the sea urchin and 18 more than in human. This is one of the most remarkable aspect of *C*. *gigas* kinome. A phylogenetic analysis of RGC protein kinases was performed to better characterize this group in *C*. *gigas* (Fig 2). We identified eight oyster-specific RGCs that differ from the expanded RGC group of *C*. *elegans* (Fig 2) and correspond to a single homolog in sea urchin. As in *C*. *gigas*, RGC kinases are also particularly abundant in *C*. *elegans* (27 members) and this particularity is not observed in other species (Fig 2). Interestingly, in *C*. *elegans*, RGC kinases have been associated with a worm-specific sensory receptor system [70] involved in alkalinity sensing [71], olfaction and odor sensing [72]. *C*. *elegans* RGCs have also been described as salt receptor proteins [73]. In *C*. *gigas*, little is known about the function of RGCs. In the American oyster *Crassostrea virginica*, the peptides binding RGCs have been characterized in gills [74] [75], where their expression levels were reduced under low salinity [76]. The high number of RGC protein kinases could be an adaptation of *C*. *gigas* living in a highly dynamic marine environment. It would therefore be interesting to determine whether the RGC group also plays a role in the oyster sensory receptor system.

### TK group

In *C*. *gigas*, the TK group contains 70 ePKs representing 19% of its kinome. Evolutionarily, this group appears to be the youngest of the kinase groups, as it is absent in plants and unicellular organisms like the amoeba *Dictyostelium discoideum* or the yeast *S*. *cerevisiae* [16] [77]. Each TK family is classified either as a membrane receptor (21 families) or as a cytoplasmic tyrosine kinase (11 families) [77]. The *C*. *gigas* kinome contains 40 TK receptor and 30 cytoplasmic TK (S1 Table). These are key components for the relay of extracellular signals into the cell.

TK receptors are involved in various biological processes, including growth, development and immunity [78]. In *C*. *gigas*, nine TK families exist as single isoforms (Ret, insR, Ryk, Sev, CCK4, Trk, LmR, VEGFR, PDGFR), including insulin-related peptide receptors involved in growth regulation [79] and EGFR. The EGFR signaling module has been highly conserved throughout the course of evolution [80]. EGFR is a cell-surface receptor that plays key roles in growth and cellular proliferation, whose function is dependent on the diversity of EGFR ligands, such as epidermal growth factor or transforming growth factor α [81]. EGFR activates several signal transduction cascades that can regulate DNA synthesis, cell proliferation, differentiation, adhesion, migration and apoptosis and in mammals, EGFR contributes to the maturation of epithelial tissues, axon regeneration, wound repair and regeneration. In *C*. *elegans*, the EGFR network plays a central developmental role, determining the fate of several types of cells [81]. In Drosophila, a unique EGRF participates in gametogenesis, segmentation, wing and eye development [82]. In *C*. *gigas*, the ligand binding domain of the gene encoding EGFR exhibits a poor sequence similarity with human EGFR [83]. Based on its expression level in *C*. *gigas* and functional analysis, both in *C*.*gigas* and in a mouse cell line, it was demonstrated that *C*. *gigas* EGFR plays a role in cell migration during wound healing of mantle and conserves the ability to activate cell proliferation [83].

Cytoplasmic TKs are involved in oxidative, temperature or osmotic regulation [84] [85] [86]. In our classification, five TK were considered as TK-unique in *C*. *gigas* based on comparisons with ePK domains from other species. In this study, we highlighted the fact that *C*. *gigas* shares an expansion of the Src family with the sea urchin. In *S*. *purpuratus*, this is required for egg activation [87] and was considered to be echinoderm-specific [17]. Like the sea urchin and Drosophila, *C*. *gigas* lacks the Axl kinase receptor that is involved in hematopoiesis in mammals [88]. In the *C*. *gigas* kinome, the most represented TK subfamily is FERs, with eight members, whereas the sea urchin and Drosophila have just a single member and the worm, in contrast, possesses 38 FERs. In *C*. *elegans*, FRK1 is a FER that is essential for morphogenesis and differentiation of the epidermis during embryonic development [89]. The putative role of FER in oyster development remains to be investigated in *C*. *gigas*.

### TKL group

The TKL group contains 40 ePKs representing 10% of the *C*. *gigas* kinome, which is similar to Drosophila, sea urchins and humans. Four TKL were classified in unique families based on domain comparisons with ePK domains from other species. The seven major families of the TKL group that have been identified in other species are present in the oyster kinome. MLK (Mixed Lineage Kinases) and RAF families are known to be sensitive to a wide range of stressors and are involved in MAPK signaling [90]. MLKL subfamilies in *C*. *gigas* are present as single homologs. Six members of LRRK (Leucine rich repeat kinases) were found in *C*. *gigas*, three in the sea urchin and one in *C*. *elegans*. In Drosophila and *C*. *elegans*, overexpression of LRRK induced neurodegeneration and modulated mitochondrial function [91] [92] [93]. In *C*. *gigas*, functional studies were performed on several TKL in the transforming growth factor β (TGF-β) pathway. Some receptors were characterized (activin-like receptors) [94] [95], including the TGF-β receptor [96] and the Bone Morphogenetic Protein Receptor (BMPR1) [97]. The role of the TGF-β signaling pathway was also described in germinal cell proliferation [98] [99] and immunity [100].

### STE group

Twenty-nine protein kinases were classified in the STE group in the *C*. *gigas* kinome. This group contains the components of the cellular signal transduction upstream of MAPK and includes three families: STE20 (MAP4K), STE11 (MAP3K) and STE7 (MAP2K). Generally, MAP4K activates MAP3K, which activates MAP2K, which finally activates MAPK [101]. The MAPK signaling pathway is crucial in eukaryotes for response to stress and signaling into the cell [102]. The 11 subfamilies of STE20 and five subfamilies of STE11 existing in metazoans are present in the *C*. *gigas* kinome, as in the sea urchin. In contrast to humans, members of the STE11 family are present as single orthologs, except for MEKK4 which has two orthologs in *C*. *gigas*. Sea urchins and oysters have the same number of STE7 protein kinases (MEK3, MEK4, MEK5 and MEK7), known to be dual specificity protein kinases because they phosphorylate their target MAPK on both the threonine and tyrosine residues. Surprisingly, the MEK5/ERK5 signaling pathway was considered to be secondarily lost by protostomes [103]. Here, we show that *C*. *gigas* possesses MEK5 (STE group) and ERK5 (CMGC group), suggesting that the MEK5/ERK5 signaling pathway exists in Lophotrochozoa and is therefore not deuterostome-specific. The existence of all the components of the MAPK signaling pathway in *C*. *gigas* may reflect its ability to be receptive and cope with environmental factors as reported in *C*. *elegans* [11]. Moreover, as suggested for the sea urchin and *C*. *elegans* [17] [18], the Pacific oyster should be a good species for investigating MAPK pathways, as the absence of redundancy would simplify functional studies.

### The “Other” group

The “Other” group includes 77 protein kinases and constitutes the largest group in *C*. *gigas* (21% of the kinome), as in the sea urchin (26%) and Drosophila (19%) (Table 1). Similarly to other species, their ePK domains do not fit into any of the other major groups described above. Various protein kinases, such as Mos (Moloney murine sarcoma kinase), SgK071 (serine/threonine Kinase-like domain containing 1) and Topk (Lymphokine-activated killer T-cell-originated protein kinase), were not found by HMM searches, but were identified using BLAST searches. However, *C*. *gigas* lacks the KIS (Kinase Interacting Stathmin), a kinase involved in advanced functions of the nervous system [104]. In the Other group, some families are especially well represented, such as the IKK (Inhibitor of nuclear factor Kappa B Kinase) with 11 members. They share structural and functional properties with their mammalian homologs and play a central role in cell signaling through Nuclear Factor-Kappa B (NF-Kappa B) [105]. NF-Kappa B is involved in the toll-like receptor pathway in innate immunity in oysters and other bivalve mollusks [3]. We also identified the Protein Kinase R (PKR) in this group, which is involved in the antiviral response [106]. The NEK (NIMA-related serine/threonine Kinase) family is abundant, with 19 members in the *C*. *gigas* kinome as opposed to 11 (NEK1 to NEK11) in humans. In vertebrates, several NEK have an important role in controlling the cell cycle by contributing to the establishment of the microtubule bundle [107]. They are also important for cellular repair and recovery from DNA damage [108] [109]. This high number of NEKs might reflect an adaptation of oysters living in a highly dynamic marine environment to cope with DNA damage.

### Atypical protein kinases

Regarding the atypical protein kinases, it was surprising to discover that *C*. *gigas* had fewer members than other species studied in kinome comparisons. For example, the Pacific oyster has nine aPKs, whereas 15 are present in yeast, 17 in Drosophila, 20 in the worm, 24 in the sea urchin and 40 in humans. This group contains a number of lipid, sugar, and other small-molecule kinases. Indeed, the oyster atypical kinases were assigned to the alpha, RIO or PIKK (Phosphatidyl inositol 3’ kinase-related kinase) families. These proteins play a role in DNA repair and cell-cycle progression, but their functions in marine invertebrates remain unknown. The atypical protein kinases are now classified in the PKL (Protein Kinase-Like) group and share common structural features with protein kinases.

### Expression of protein kinases during development

**Fig 3:**
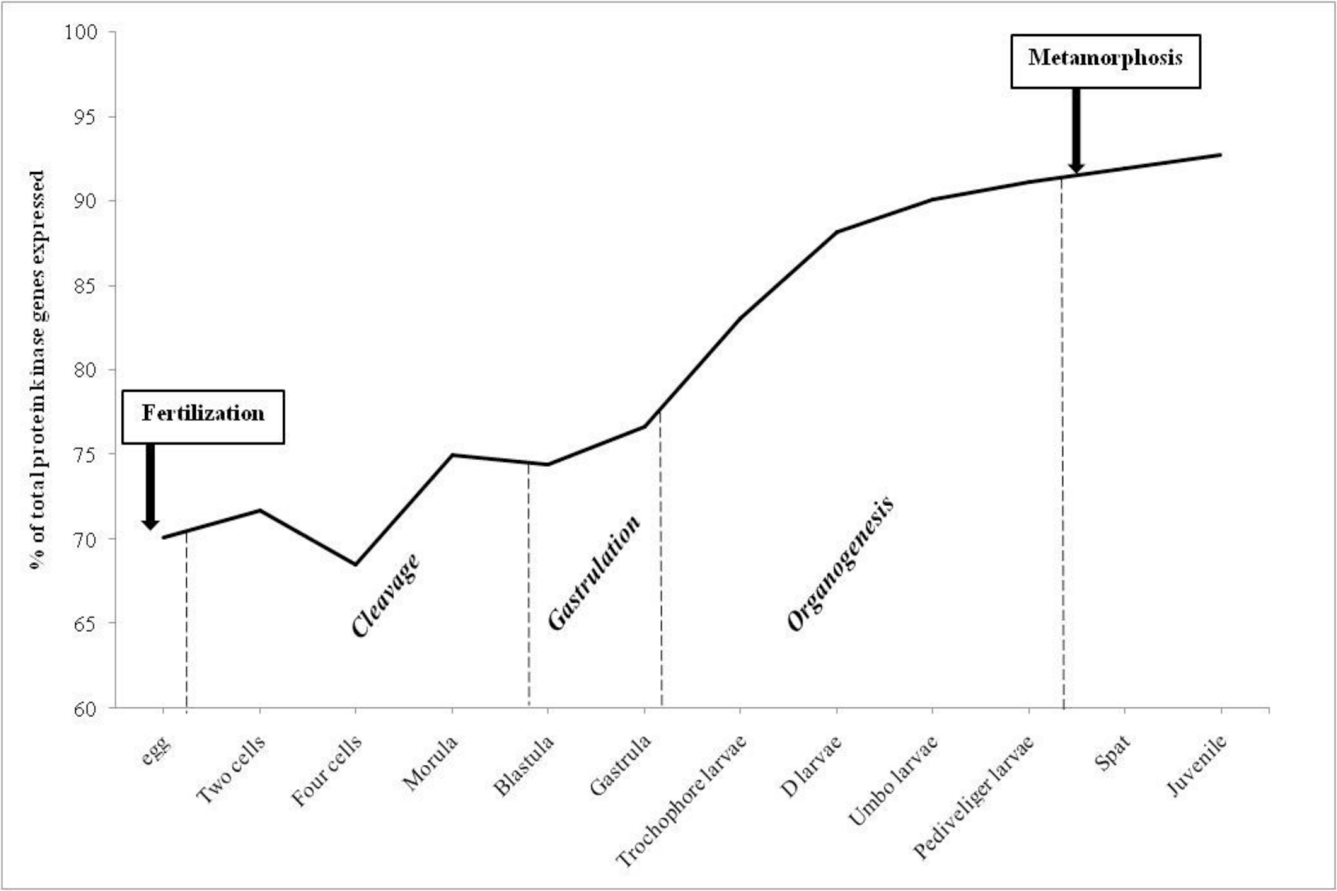
Number of protein kinases genes expressed during *Crassostrea gigas* development. Number of ePK and aPK genes expressed during *Crassostrea gigas* development (% of total 371 protein kinases). The development stages were grouped according to the following: cleavage begins from the two cell stage to blastula, gastrulation is from blastula to gastrula stages. Genes with expression values < 1 RPKM were considered to be non-expressed.

Transcriptomes from developmental stages in *C*. *gigas* were obtained from RNA-seq (49 bp single-end Illumina reads) on a total of 250,000 zygotes maintained at 26°C and salinity around 30 ppm [5]. They provide valuable resources for studying protein kinase expression, in order to determine which kinases could be involved in the differentiation of cell types from egg to juvenile. These transcriptomes were obtained from 38 biological samples representing three major developmental processes: cleavage, gastrulation and organogenesis. In this analysis, a threshold of 1 RPKM was used to classify genes as expressed or not (S1 Table). Our results show that more than 70% of the kinome was expressed during early development in *C*. *gigas* (Fig 3). Indeed, 70% of the kinome was expressed in eggs, 74% in embryos at the end of the cleavage (blastula stage), 77% at the end of the gastrulation, 83% in trochophore larvae, 88% in D-larvae, 90% for umbo larvae, 91% for pediveliger larvae, then 91% in spat and the maximum of 93% in juveniles (Fig 3). The lowest percentage of kinome was expressed at the 4-cell stage (68%; Fig 3) and might reflect a basal activity of weakly expressed genes, whose RPKM is close to the threshold of 1. From egg to metamorphosis, protein kinase genes belonging to all the classified groups in *C*. *gigas* were expressed, but not from all subfamilies (S1 Table). Not surprisingly, the kinases associated with spermiogenesis, such as TSSK in the CAMK group, were weakly expressed since maturation had not occurred either in spat nor in juveniles (S1 Table).

Embryogenesis corresponds to the process by which the embryo forms and develops. It starts with the fertilization of the egg cell and is followed by mitotic divisions, known as cleavage, leading to a late embryo called morula. From egg to juvenile, all the aPK genes are expressed, suggesting their involvement in all stages of the developing embryo. Almost all genes belonging to CMGC group, including MAPK signaling, were expressed in eggs (92%; Table 2). During cleavage, three more CMGC genes were expressed, suggesting their implication during the first mitotic divisions: two CDK7 (Cyclin-Dependent Kinase 7) and DYRK2 (Dual-specificity tYrosine phosphorylation Regulated Kinase 2). In various species including the gastropod *Haliotis asinina*, CMGC kinases including MAPK signaling are crucial in patterning and establishing axial symmetry during embryogenesis [102]. We can hypothesize that the expression of all representatives of CMGC group in *C*. *gigas* from egg to morula might reflect their conserved roles in the organization of the symmetry in oyster embryos. Gastrulation follows the cleavage stages and is characterized by cell movements resulting in a massive reorganization of the embryo from a simple spherical ball of cells, the blastula, to a multi-layered organism, the gastrula. During gastrulation, many of the cells at or near the surface of the embryo move to a more interior location. We identified several protein kinase genes belonging to different ePK groups that start to be expressed during gastrulation: PKCα, LRRK, PKG, HUNK, BRSK, DCAMKL, QIK, MEKK2 and GCN2 (S1 Table). They may be involved in convolution and differentiation of the cells into different dermal layers. In the frog *Xenopus laevis*, the different isoforms of protein kinase PKC are associated with Wnt signaling, leading to convergent extension movements [110]. LRRK2 is also involved in Wnt signaling in vertebrates [111]. PKG (c-GMP dependent protein kinase) is expressed during gastrulation of the medaka fish *Oryzias latipes* and is necessary to maintain embryo development by phosphorylating targets of SHH (Sonic Hedgehog), a crucial pathway for embryogenesis in vertebrates [112]. These pathways linked with gastrulation processes might thus be conserved between vertebrates and invertebrates.

**Table 2:** Number and percentage of ePK and aPK genes expressed during *Crassostrea gigas* development.

		Expressed genes								
Group	Total genes	Egg	Cleavage	Gastrulation	Trochophore larvae	D larvae	Umbo larvae	Pediveliger larvae	Spat	Juvenile
**AGC**	**28**	25	25	28	28	28	28	28	28	28
		89%	89%	100%	100%	100%	100%	100%	100%	100%
**CAMK**	**51**	36	38	39	43	43	42	43	43	44
		71%	75%	76%	84%	84%	82%	84%	84%	86%
**CMGC**	**39**	36	39	39	39	39	39	39	39	39
		92%	100%	100%	100%	100%	100%	100%	100%	100%
**CK1**	**6**	5	6	6	6	6	6	6	6	6
		83%	100%	100%	100%	100%	100%	100%	100%	100%
**RGC**	**23**	4	5	6	12	15	16	18	15	13
		17%	22%	26%	52%	65%	70%	78%	65%	57%
**TK**	**70**	42	44	43	48	53	59	59	63	63
		60%	63%	61%	69%	76%	84%	84%	90%	90%
**TKL**	**40**	25	27	30	31	36	37	36	39	39
		63%	68%	75%	78%	90%	93%	90%	98%	98%
**STE**	**28**	22	24	25	27	27	28	28	27	28
		79%	86%	89%	96%	96%	100%	100%	96%	100%
**Other**	**77**	56	59	61	65	71	70	72	72	75
		73%	77%	79%	84%	92%	91%	94%	94%	97%
**aPK**	**9**	9	9	9	9	9	9	9	9	9
		100%	100%	100%	100%	100%	100%	100%	100%	100%

The development stages were grouped according to the following: cleavage begins from the two cell stage to blastula, gastrulation is from blastula to gastrula stages.

In *C*. *gigas*, after gastrulation, organogenesis starts with the development of the larva (trochophore, D-shape, umbo and pediveliger) involving cell reorganization until metamorphosis. Some protein kinase genes start to be expressed and belong to several groups: the CAMK group, with CASK (Calcium/calmodulin-dependent Serine protein Kinase) and MLCK (Myosin Light-Chain Kinase), the STE group with YSK (Yeast Sps1/Ste20-related Kinase 4) and MKC (Metastatic Kidney Cancer), and some members of NEK families in the Other group (S1 Table). Because their mRNAs are expressed from the first steps of the cleavage, MLCK and NEK might belong to the regulation of the early development in *C*. *gigas*. MLCK was shown to be crucial to larval settlement of the intertidal barnacle *Balanus amphitrite*, by modulating muscle contraction and motility of larvae [113]. MLCK is also involved in axon pathway formation in Drosophila embryos [114]. NEK proteins were identified in mammals as necessary for the development of the nervous system [107].

Metamorphosis is an important step, transforming larvae into spat with a reorganization of most larval organs to form a juvenile oyster with its definitive organs. We identified 3 protein kinase genes belonging to the TK group, Syk (Spleen tyrosine kinase), Tie (tyrosine kinase with immunoglobulin-like and EGF-like domains) and Csk (C-Src kinase) that start to be expressed between the pediveliger larval stage and spat (S1 Table). We can hypothesize that these tyrosine kinases might participate in the regulation of metamorphosis. Indeed, in the bryozoan *Bugula neritina* and the barnacle *Balanus amphitrite*, a tyrosine kinase inhibitor was shown to prevent metamorphosis [115].

### Expression of protein kinases under environmental stressors

We performed an *in silico* analysis to identify down- and up-regulated kinases genes in oysters subjected to 8 different potential sources of stress: temperature, salinity, exposure to air, and to five metals (cadmium, copper, mercury, lead and zinc) [5]. Among the 371 genes encoding protein kinases, we found that 177 genes (48% of the *C*. *gigas* kinome) were differentially expressed under at least one factor relative to the compared condition (Table 3). This corresponds to around 3% of the 5,844 genes identified from transcriptome datasets that were modulated (up or down-regulated) by these factors [5].

**Table 3:** Number and percentage of ePK and aPK genes up- and down-regulated under environmental stressors.

		**Differentially expressed genes under stressors**															
Group	Total genes	Exposure to air		Temperature		Salinity		Cadmium		Copper		Mercury		Lead		Zinc	
		Up	Down	Up	Down	Up	Down	Up	Down	Up	Down	Up	Down	Up	Down	Up	Down
**AGC**	**28**	1	3	0	0	0	1	0	0	0	0	0	0	0	0	2	1
		4%	11%	**-**	**-**	-	4%	**-**	-	**-**	-	**-**	-	**-**	-	7%	4%
**CAMK**	**51**	1	15	2	5	2	3	0	1	0	0	0	2	1	0	2	3
		2%	29%	4%	10%	4%	6%	-	2%	**-**	-	-	4%	2%	-	4%	6%
**CMGC**	**39**	1	16	3	5	2	0	0	1	0	1	0	1	1	1	3	0
		3%	41%	8%	13%	5%	-	-	3%	-	3%	-	3%	3%	3%	8%	-
**CK1**	**6**	0	3	0	0	0	0	0	0	0	0	0	0	0	0	0	0
		-	50%	**-**	-	**-**	-	**-**	-	**-**	-	**-**	-	**-**	-	**-**	-
**RGC**	**23**	1	5	1	0	0	0	1	0	0	0	0	1	0	0	2	0
		4%	22%	4%	-	**-**	-	4%	-	**-**	-	-	4%	**-**	-	9%	-
**TK**	**70**	11	12	9	3	5	2	5	1	2	1	5	1	6	0	17	4
		16%	17%	13%	7%	7%	3%	7%	1%	3%	1%	7%	1%	9%	-	24%	6%
**TKL**	**40**	1	9	1	4	5	1	3	0	0	4	0	2	3	1	7	4
		2%	23%	3%	10%	13%	2%	8%	-	-	10%	-	5%	8%	3%	18%	10%
**STE**	**28**	2	4	0	0	0	0	0	1	0	1	0	0	0	0	4	0
		7%	14%	**-**	-	**-**	-	-	4%	-	4%	**-**	-	**-**	-	14%	-
**Other**	**77**	0	23	5	12	7	6	2	6	0	0	4	4	3	12	12	5
		-	30%	6%	16%	9%	8%	3%	8%	**-**	-	5%	5%	4%	16%	16%	6%
**aPK**	**9**	0	2	0	2	0	2	0	0	0	2	0	0	0	1	2	0
		-	22%	-	22%	-	22%	**-**	-	-	22%	**-**	-	-	11%	22%	-
**Total**	**371**	**18**	**92**	**21**	**31**	**21**	**15**	**11**	**10**	**2**	**9**	**9**	**11**	**14**	**15**	**51**	**17**

Transcriptome data were obtained by RNA-seq, normalized, and differentially expressed genes were detected using a referenced statistical method constructed based on a Poisson distribution [5]. Eight types of stressors are shown (exposure to air, temperature, salinity, and to 5 metals: cadmium, copper, mercury, lead and zinc).

**Fig 4:**
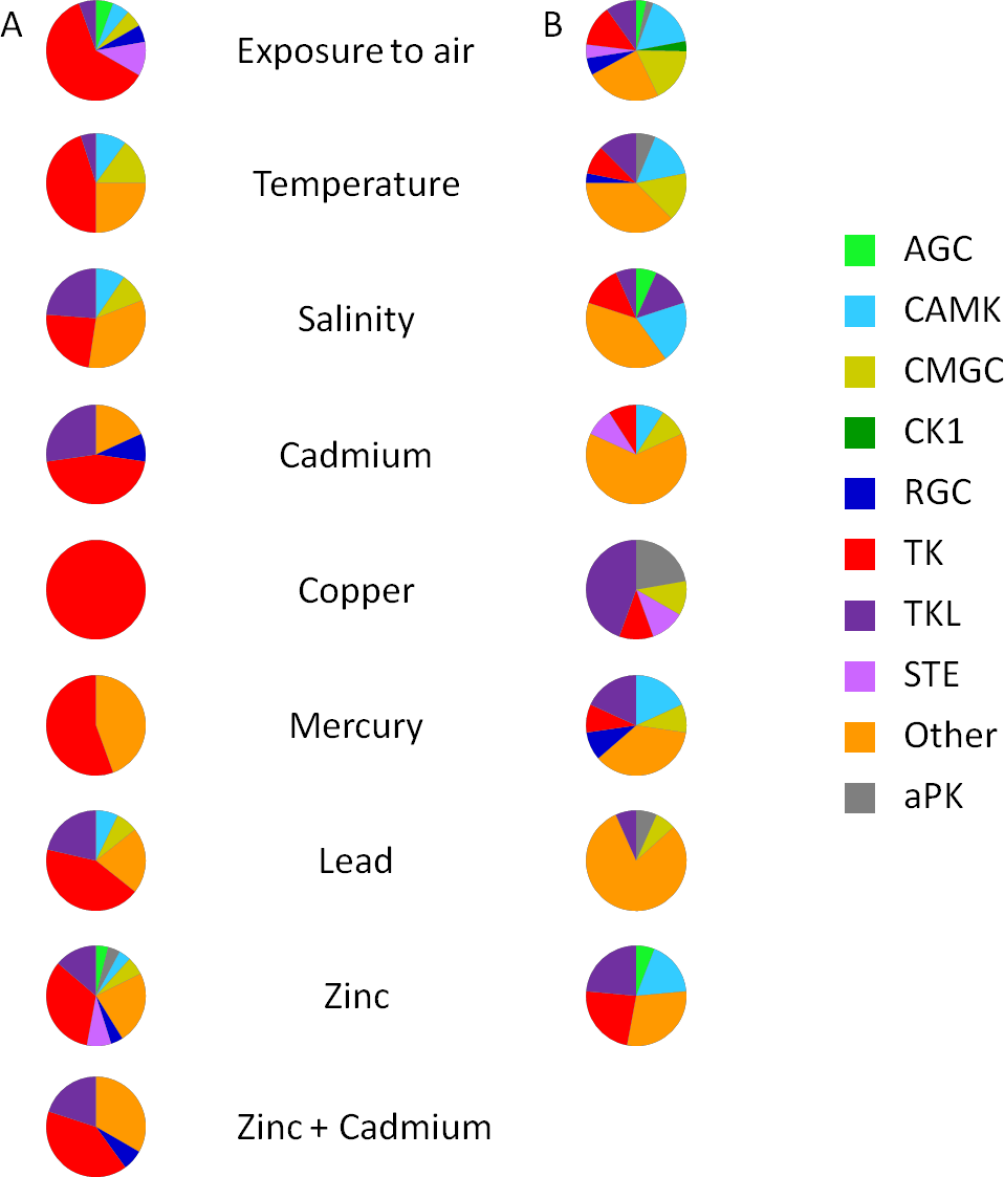
Group-level comparison of ePK and aPK genes up and down-regulated under environmental stressors. (A) Up-regulated protein kinases. (B) Down-regulated protein kinases. Pie charts depict the proportion of the protein kinases group, and the total number of pie is presented in Table 3. The absence of a pie chart means no regulation was observed.

Exposure to air is a serious stressor, causing hypoxia and leading to a decrease of ATP concentration in the hemolymph [116]. Among the different potential sources of stress, air exposure affected the expression of the largest number of protein kinase genes (110), corresponding to 30% of the oyster kinome (Table 3). Interestingly, three genes of the CK1 group were under-expressed following exposure to air (Table 3) without being affected by any other stressors (Fig 4). The highest numbers of differentially expressed genes under exposure to air were found in the TK (11 up-regulated, 12 down-regulated) and Other (23 down-regulated) groups (Table 3). Given the expected roles of TK and NEKs (in the Other group), the regulation of cell growth, differentiation, proliferation, cell-cycle or apoptosis might be modified by exposure to air.

Thermal stress triggered changes in the expression of 53 protein kinase genes (Table 3), corresponding to 14% of the *C*. *gigas* kinome and 7% of the 776 differentially expressed genes identified in the transcriptome data [5]. The response of *C*. *gigas* to thermal stress includes inhibition of apoptosis, stabilization of protein conformation and protein refolding [117]. Several genes in the TK and Other groups were up or down–regulated and we can hypothesize that the functions of these kinases can be modulated by a temperature stress in oysters. The PI3K (Phosphatidylinositol-4,5-bisphosphate 3-Kinase)/AKT/mTOR (mechanistic target of rapamycin) pathway was shown to be involved in the oyster’s response to chronic thermal stress during three months [118]. In contrast, we observed that none of the genes from the AGC group, including AKT kinase, responded to 12 hours or 7 days of thermal stress. Similarly, we showed that none of the mRNA encoding STE kinases were differentially expressed after thermal stress, although MAPK signaling was previously shown to be activated for signal transduction in stress conditions [57] [58] [101]. Actually, the absence of gene regulation might not necessarily reflect the absence of function, since protein kinases are known to be mostly activated at the post-translational level.

Estuaries are characterized by variations in salinity due to rainfall and tides. Organisms like *C*. *gigas* are subjected daily to these variations and have developed mechanisms to adapt their behavior by closing their shells and ceasing to feed when exposed to low-salinity water [119]. Variations in salinity changed the expression of 35 protein kinase genes (Table 3), corresponding to 9.5% of the *C*. *gigas* kinome and 3% of the 1024 differentially expressed genes identified in the transcriptome data [5]. Based on our classification, we showed that protein kinase genes differentially expressed under the salinity constraint were mainly associated with the regulation of metabolism, cytoskeletal organization, and immune response (S1 Table). In the CAMK group, hyposalinity changed the expression of 3 MLRK genes (2 up-regulated; 1 down-regulated) (S1 Table). MLRK are known to be myosin light chain kinases associated with the passive elasticity of muscle. Our results suggest reorganization of cytoskeletal components during hyposalinity. Five kinases from TK group (Csk, Met, Eph and Ror, Fer) were up-regulated in response to hyposalinity (Table 3), indicating that variations of salinity could modulate the cell adhesion and communication, signal transduction and cytoskeleton organization in *C*. *gigas*. In TK group, hyposalinity up-regulated 1 Fer (S1 Table), a protein kinase known to be involved in signaling and regulation of cell-cell interactions [120], as already observed in *C*. *gigas* under salinity stress [121].

In the Other group, the gene encoding PKR (protein kinase R) was up-regulated in response to hypersalinity and down-regulated in response to hyposalinity (S1 Table). In *C*. *gigas*, over-expression of the PKR gene has been associated with a protective antiviral immune response against Ostreid herpesvirus (OsHV-1 μvar), induced by polyinosinic: polycytidylic acid (Poly I: C) injection [106] [122]. In mammals, the activation of PKR is one of the mechanisms permitting early blocking of viral replication via inhibition of protein synthesis [123] and activation of autophagy [124]. The PKR gene in *C*. *gigas* is homologous to vertebrate ISG (interferon stimulated gene) and plays a role in pathogen recognition and activation of innate immunity [122]. Taken together, modulation of PKR by salinity might indicate that salinity could have an impact on the ability of *C*. *gigas* to resist to viral infections.

Oysters living in coastal environments that suffer from human expansion may also be exposed to anthropogenic contaminations, such as heavy metals [125]. Heavy metal exposure changed the expression of 96 mRNA encoding protein kinases (S1 Table), corresponding to 26% of the *C*. *gigas* kinome and 9% of the 1024 differentially expressed genes identified in the transcriptome data [5]. With the exception of the CK1 group, genes encoding kinases in all ePK groups responded to heavy metal exposure (Fig 4). Heavy metal exposure modulated the expression of genes that mainly belong to TK, TKL and Other groups (Table 3; Fig 4). Most of these TK genes were up-regulated, and we can hypothesize that changes in TK expression could reflect that heavy metals can interfere on growth hormone regulation, as observed in fish [126].

## Conclusion

The characterization of the *C*. *gigas* kinome and the identification of differentially expressed protein kinases was made possible thanks to the recently published oyster genome and a rich transcriptome datasets. The *C*. *gigas* kinome might have an original evolutionary position, because the number, classification and distribution of protein kinases is closer to deuterostomes (sea urchin and human) than to protostomes (nematode and fly). Kinase gene redundancy in the *C*. *gigas* kinome concerns about 30% of the genes. The lack of redundant protein kinases isoforms in several groups suggests that the Pacific oyster *C*. *gigas* could be a good species for the development of functional research dealing with protein kinases, for example, to study developmental processes, given that most protein kinases are expressed during the first stages of embryogenesis. Here we also identified the kinases that are mobilized in the Pacific oyster to deal with changes in temperature or salinity, with exposure to air, and after contamination by heavy metals, as well as during development. We provide new insights into the key pathways that may be crucial for adaptation to life in a highly dynamic environment.

## Supporting Information

S1 Table Identified *Crassostrea gigas* protein kinases. Genbank accession number, classification (group/family/subfamily), query definition, best hit name, percentage of identity compared to other species with corresponding E-values, embryonic expression and differentially expression induced by stressors. Y: yes. U: up-regulation. D: Down-regulation.
(XLSX)

S1 Text Zhang et al. (2012). (PDF)
